# Supplementary material for: Biased signaling downstream of epidermal growth factor receptor regulates proliferative versus apoptotic response to ligand
Source: Cell Death Dis. 2018 Sep 24;9(10):976. doi: 10.1038/s41419-018-1034-7 (PMC6155319; doi:10.1038/s41419-018-1034-7)
Supplement: Supplementary file 1 — Supplemental Tables 1–3 [file 41419_2018_1034_MOESM1_ESM.docx]

**Supplementary Table 1**

| **Target** | **TRC#** | **Sequence (mature antisense)** |
| --- | --- | --- |
| mSTAT1#23 | TRCN0000054923 | ATTCTCTGGTATGTTCTCGGC |
| mSTAT1#24 | TRCN0000054924 | TAAGAGAGTGAAGTTCTTCGG |
| mSTAT1#25 | TRCN0000054925 | AAACGAGACATCATAGGCAGC |
| mSTAT1#26 | TRCN0000054926 | AATATCTGGGAAAGTAACAGC |
| mSTAT1#27 | TRCN0000054927 | ATCAGAGTGTTCTGAGTGAGC |

**Supplementary Table 2**

| **Name** | **Target** | **Concentration** | **Supplier** |
| --- | --- | --- | --- |
| trametinib | MEK1/2 | Indicated | Selleckchem |
| gefitinib | EGFR | 500 nM | Selleckchem |
| Erlotinib | EGFR | Indicated | Selleckchem |
| NLS-gefitinib | EGFR | 1μM | Davisson Lab |
| leptomycin B | CRM1 | 20 nM | Cell Signaling Technologies |
| EGF | - | Indicated | GoldBio |
| IL-6 | - | 20 ng/ml | GoldBio |

**Supplementary Table 3**

| **Antibody** | **Dilution** | **Supplier** |
| --- | --- | --- |
| pSTAT1(Y701) | 1:1000 | Cell Signaling Technologies |
| tSTAT1 | 1:1000, 1:100 (I.F.) | Cell Signaling Technologies |
| pEGFR(Y845) | 1:1000 | Cell Signaling Technologies |
| tEGFR | 1:1000 | Cell Signaling Technologies |
| EGFR | 1:100 (I.F) | Santa Cruz Biotechnologies |
| pERK1/2 | 1:2000 | Cell Signaling Technologies |
| tERK1/2 | 1:2000, 1:100 (I.F) | Cell Signaling Technologies |
| β-actin | 1:1000 | Santa Cruz Biotechnologies |
| Lamin A/C | 1:500 | Santa Cruz Biotechnologies |
| β-tubulin | 1:1000 | Developmental Studies Hydbridoma Bank |
