# Supplementary figures and images for: Biased signaling downstream of epidermal growth factor receptor regulates proliferative versus apoptotic response to ligand
Source: Cell Death Dis. 2018 Sep 24;9(10):976. doi: 10.1038/s41419-018-1034-7 (PMC6155319; doi:10.1038/s41419-018-1034-7)

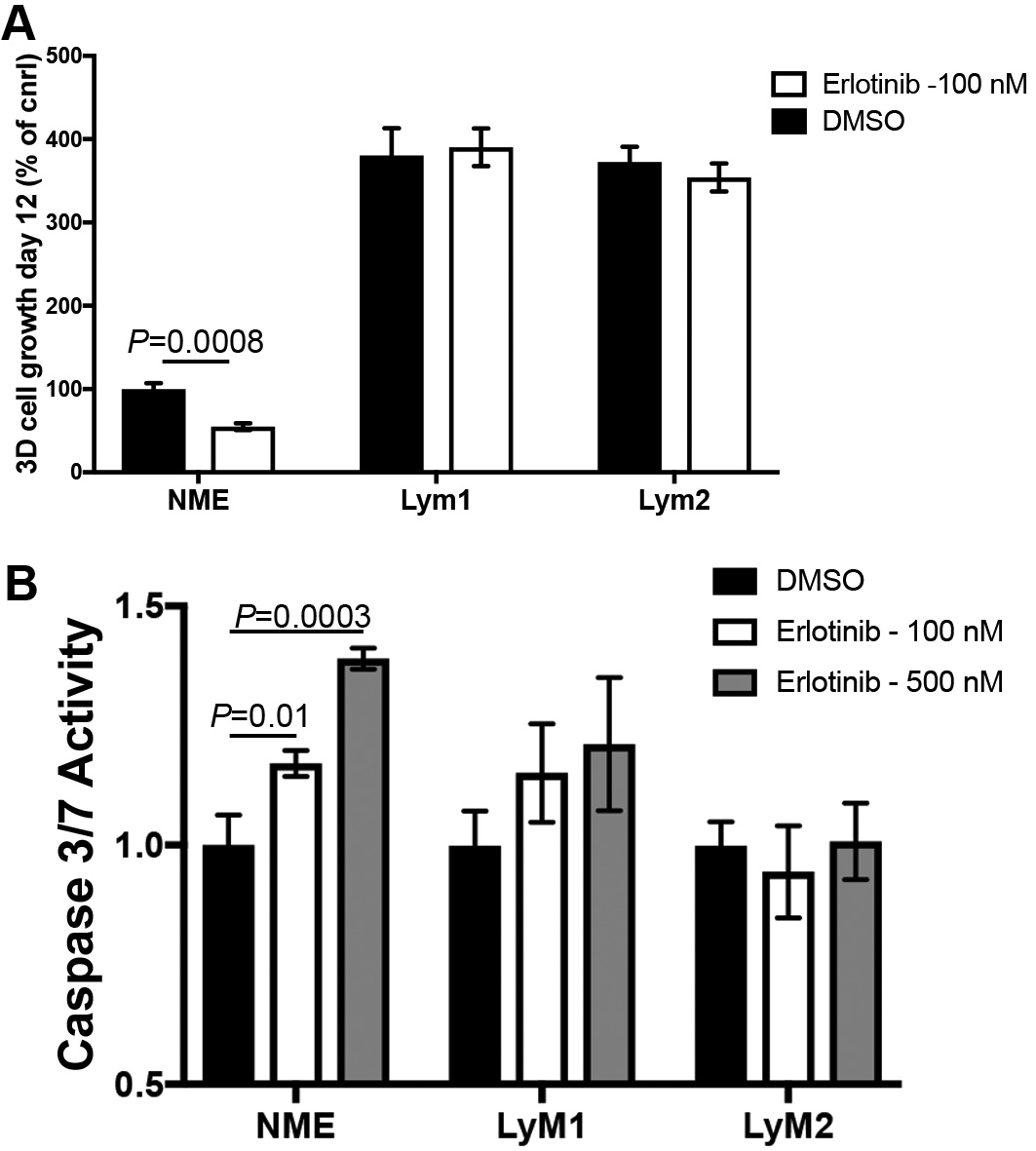

Supplement: Supplementary file 2 — Supplemental Figure 1 [file 41419_2018_1034_MOESM2_ESM.jpg]

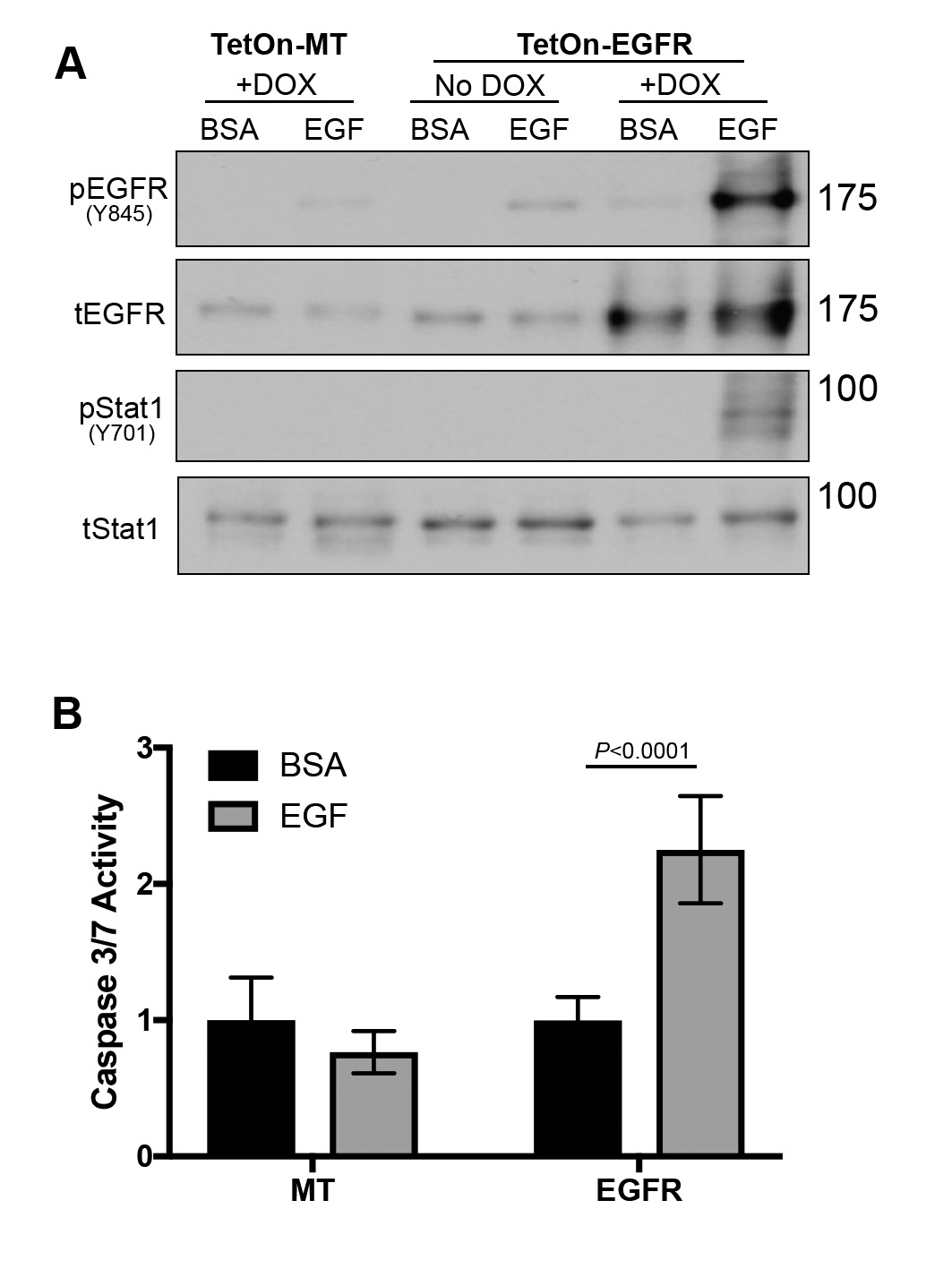

Supplement: Supplementary file 3 — Supplemental Figure 2 [file 41419_2018_1034_MOESM3_ESM.jpg]

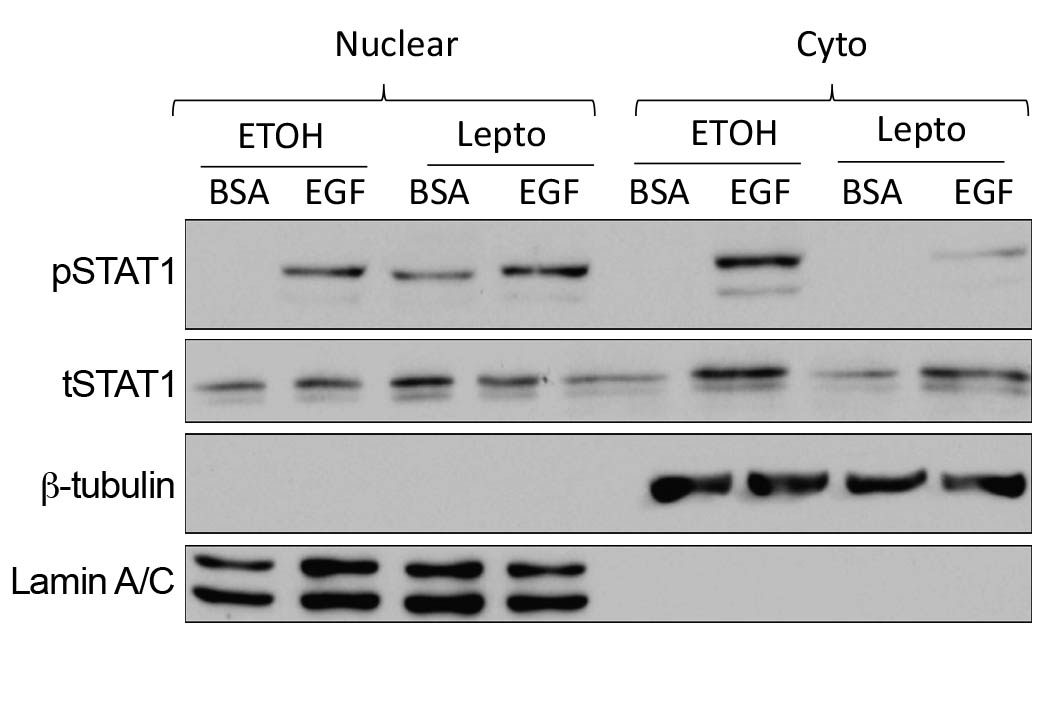

Supplement: Supplementary file 4 — Supplemental Figure 3 [file 41419_2018_1034_MOESM4_ESM.jpg]

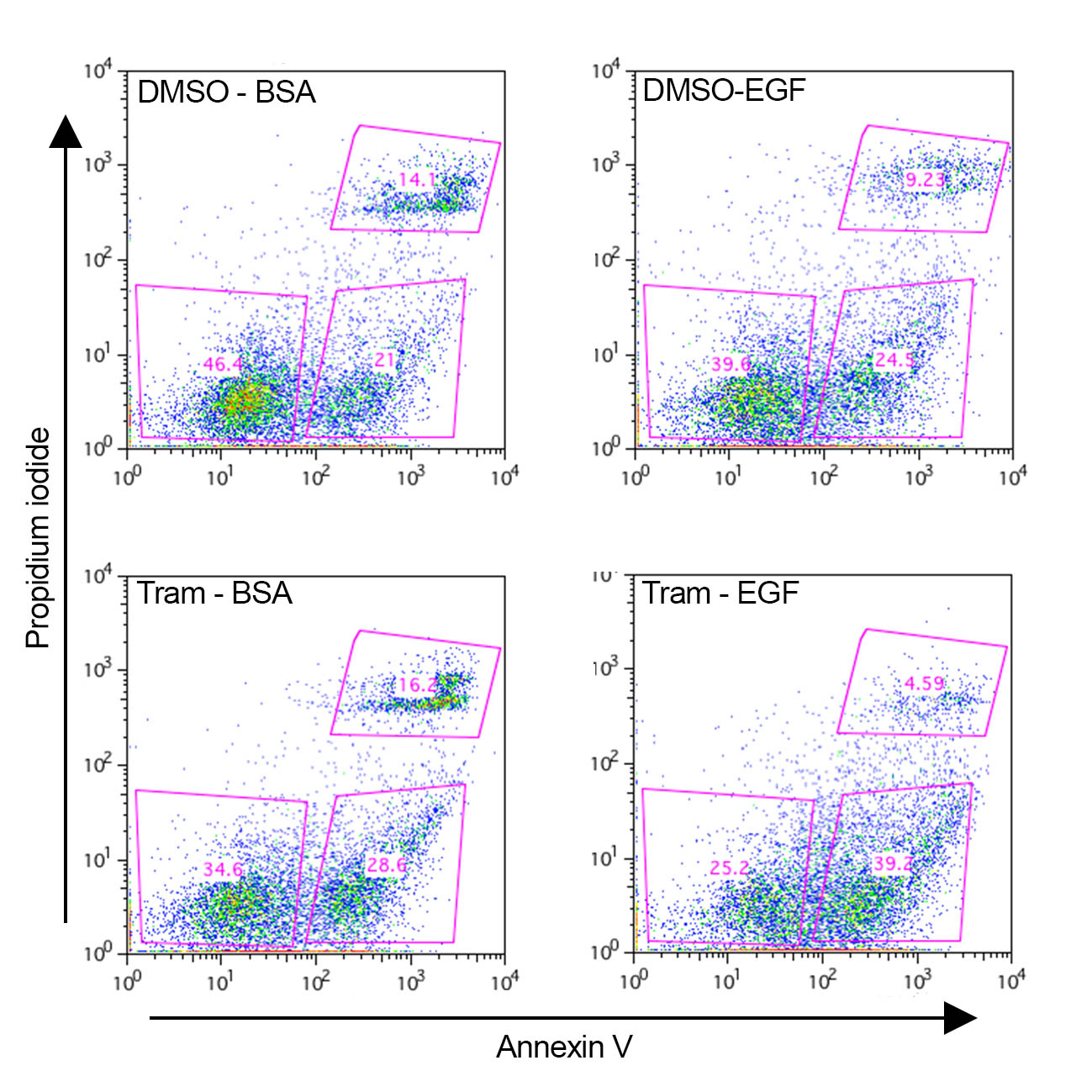

Supplement: Supplementary file 5 — Supplemental Figure 4 [file 41419_2018_1034_MOESM5_ESM.jpg]

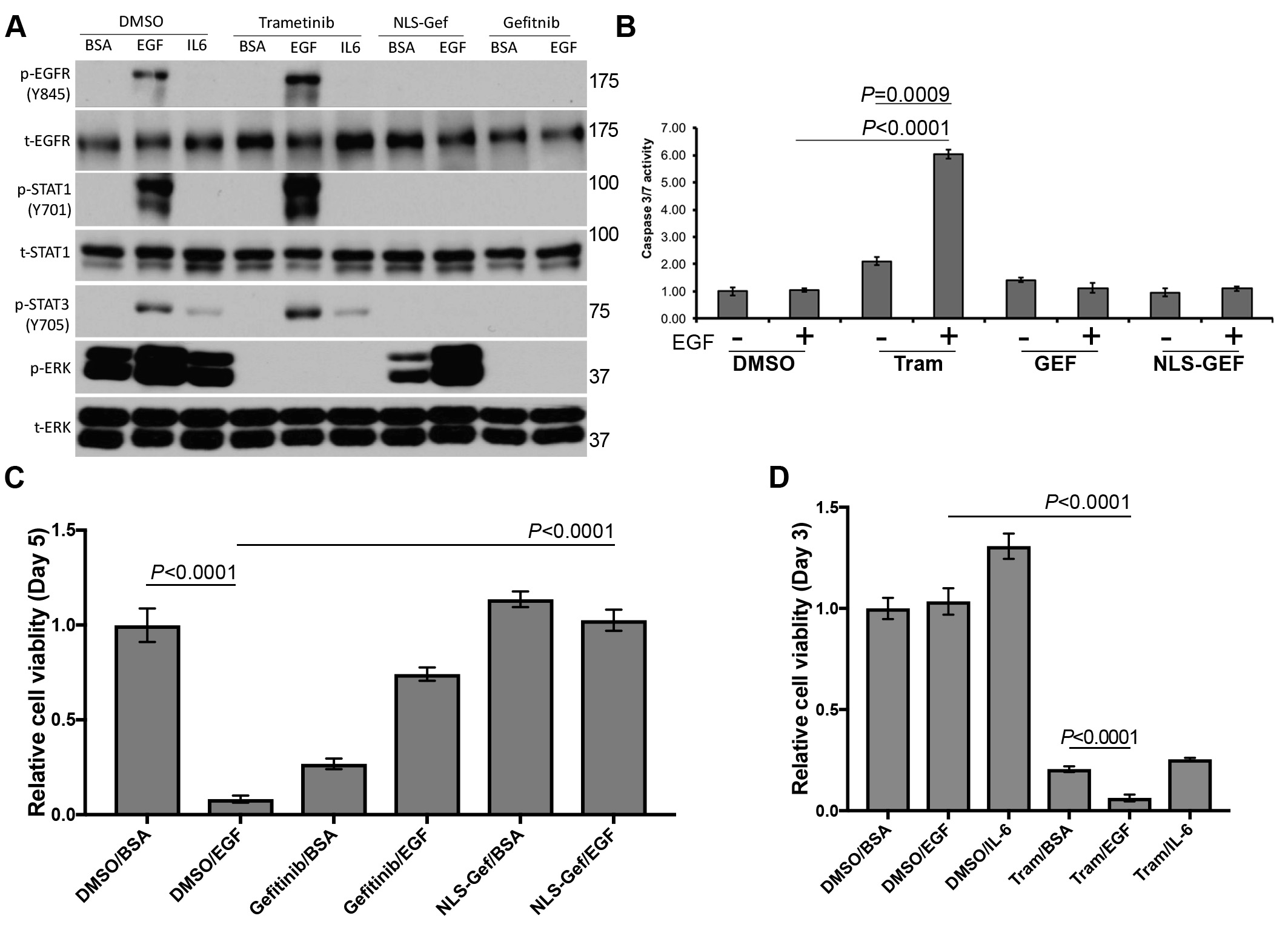

Supplement: Supplementary file 6 — Supplemental Figure 5 [file 41419_2018_1034_MOESM6_ESM.jpg]

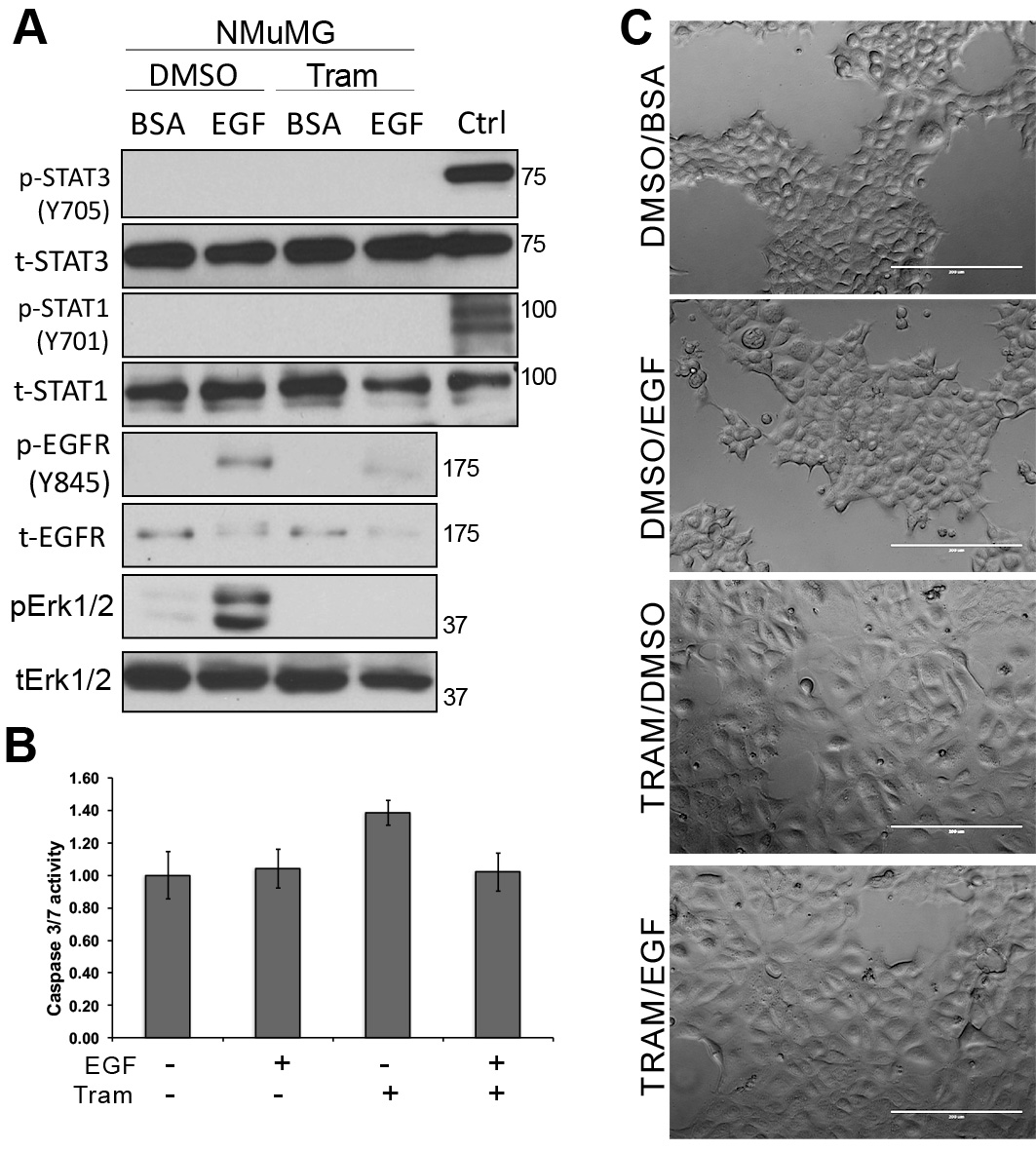

Supplement: Supplementary file 7 — Supplemental Figure 6 [file 41419_2018_1034_MOESM7_ESM.jpg]
