# Supplementary material for: Biased signaling downstream of epidermal growth factor receptor regulates proliferative versus apoptotic response to ligand
Source: Cell Death Dis. 2018 Sep 24;9(10):976. doi: 10.1038/s41419-018-1034-7 (PMC6155319; doi:10.1038/s41419-018-1034-7)
Supplement: Supplementary file 8 — Supplementary figure legends [file 41419_2018_1034_MOESM8_ESM.docx]

Supplementary Figure legends:

**Fig. S1.** EGFR-transformed lymph node metastases are resistant to inhibition of EGFR kinase activity. **(A)** EGFR transformed mammary epithelial cells (NME) and their isogeneic lymphnode-derived metastatic counterparts (Lym1 and Lym2) expressing firefly luciferase were cultured under 3D conditions in the presence or absence of the EGFR inhibitor erlotinib for 12 days at which point cellular viability was quantified by bioluminescence. **(B)** The cells described in panel A were cultured on 2D tissue plastic and treated for 24 hours with the indicated concentrations of erlotinib and subsequently analyzed for caspase 3/7 activity. Data are the mean ± SE of three separate experiments completed in triplicate, resulting in the indicated *P* values.

**Fig. S2.** Induction of EGFR in metastatic breast cancer cells is sufficient for ligand induced STAT1 phosphorylation and apoptosis. **(A)** Metastatic MCF10-Ca1a cells were constructed to express EGFR under the control of a tetracycline-induced promoter. Following a 24-hour induction with doxycycline (DOX; 1 μg/ml) these cells were stimulated with EGF (50 ng/ml) for 30 minutes and assayed for phosphorylation of EGFR and STAT1. Expression of total EGFR and STAT1 served as loading controls. **(B)** MCF10-Ca1a cells were constructed to stably express EGFR. These cells were stimulated with EGF (50 ng/ml) for 24 hours and assayed for caspase 3/7 activity.

**Fig. S3.** STAT1 is constitutively localized to the nucleus. NME cells were stimulated with EGF for 30 minutes in the presence or absence of the nuclear export inhibitor leptomycin B (Lepto). These cells were split into nuclear and cytoplasmic fractions and each fraction was assayed for the presence of total and phosphorylated STAT1. Lamin A/C and β-tubulin served as loading controls for the nuclear and cytoplasmic fractions, respectively.

**Fig. S4.** Combined treatment with Trametinib enhances EGF-induced apoptosis. NME-LM1 cells were stimulated with EGF (100 ng/ml) in the presence or absence of trametinib (Tram). Following 24 hours of treatment, these cells were trypsinized and stained with Annexin V antibodies and propidium iodide. Stained cells were analyzed by flow cytometry. The percentage of the total cell population in each gate is indicated.

**Fig. S5.** Inhibition of ERK1/2 signaling causes EGF-induced apoptosis in lung metastatic breast cancer cells. **(A)** Lung metastatic (NME-LM2) cells were pretreated with trametinib, gefitinib, or a nuclear localization sequence-gefitinib conjugate (NLS-GEF) and then stimulated with EGF (50 ng/ml) for 30 minutes. These cells were subsequently analyzed for phosphorylation of EGFR, STAT3, STAT1 and ERK1/2. BSA (0) served as a protein stimulation control and total levels of EGFR, STAT1 and ERK1/2 were assessed as loading controls. **(B)** NME-LM2 cells were stimulated with EGF (100 ng/ml) in the presence or absence of the indicated inhibitors (Tram = trametinib, GEF = gefitinib, NLS-GEF = gefitinib conjugated to a nuclear localization sequence). Twenty-four hours later cells were assessed for caspase 3/7 activity. **(B)** LM2 cells were treated with trametinib (Tram; 5 nM) in the presence or absence of EGF (50 ng/ml) or IL6 for three days at which point cell viability was quantified. **(C)** LM2 cells were stimulated with EGF (50 ng/ml) in the presence or absence of the indicated inhibitors for 5 days at which point cell viability was quantified. All data are the mean ± SE of three separate experiments completed in triplicate resulting the indicated *P* values.

**Fig. S6.** A combination of EGF and trametinib does not induce STAT1 phosphorylation or apoptosis in normal mammary epithelial cells. **(A)** Nontransformed NMuMG cells were stimulated with EGF (50 ng/ml) for 30 minutes in the presence or absence of trametinib (tram; 100 nM). These cells were lysed and analyzed for phosphorylation of STAT1, ERK1/2 and EGFR. Expression of total EGFR, STAT1 and ERK1/2 served as loading controls. **(B)** NMuMG cells were stimulated with EGF (100 ng/ml) in the presence and absence of trametinib (Tram; 100 nM) for 24 hours and these cells were assessed for caspase 3/7 activity. Data are the mean ± SE of three separate experiments completed in triplicate. **(C)** Phase contrast photomicrographs of NMuMG cells treated as described in panel B.
